# Supplementary material for: Development of chemokine network inhibitors using combinatorial saturation mutagenesis
Source: Commun Biol. 2025 Apr 3;8:549. doi: 10.1038/s42003-025-07778-6 (PMC11969024; doi:10.1038/s42003-025-07778-6)
Supplement: Supplementary file 4 — Supplementary Data 2 [file 42003_2025_7778_MOESM4_ESM.zip › SupplementaryData3/scripts/xattune_dr.tar!/./script/analysis/JK/JK_E17_CCL14_THP1_plot.pdf]

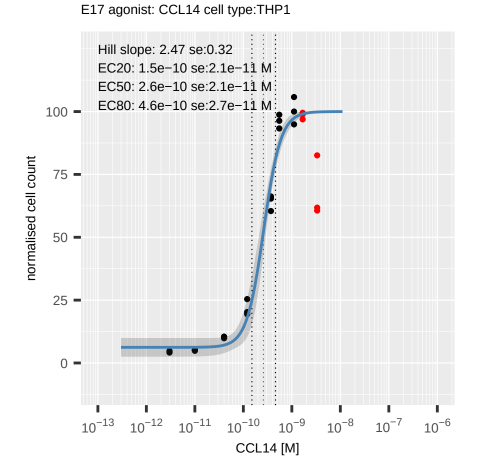

E17 agonist: CCL14 cell type:THP1
Hill slope: 2.47 se:0.32
EC20: 1.5e−10 se:2.1e−11 M
EC50: 2.6e−10 se:2.1e−11 M
EC80: 4.6e−10 se:2.7e−11 M
normalised cell count
100
75
50
25
0
10−13
10−12
10−11
10−10
10−9
10−8
10−7
10−6
CCL14 [M]
